# Iron Transport through Ferroportin Is Induced by Intracellular Ascorbate and Involves IRP2 and HIF2α

**DOI:** 10.3390/nu6010249

**Published:** 2014-01-03

**Authors:** Nathalie Scheers, Ann-Sofie Sandberg

**Affiliations:** Food Science, Division of Life Sciences, Department of Chemical and Biological Engineering, Chalmers University of Technology, Gothenburg SE-41296, Sweden; E-Mail: ann-sofie.sandberg@chalmers.se

**Keywords:** ascorbate, iron, ferroportin, IRP2, HIF2α

## Abstract

A few tightly regulated transport proteins mediate iron absorption across the intestinal epithelium. At the basolateral border of intestinal cells there is one identified transporter, ferroportin, for the transfer of intracellular iron to the vascular system. Here, we investigate the effects of ascorbate (vitamin C) on the regulation of ferroportin in human intestinal Caco-2 cells using ELISA and Western Blot analyses. The results indicate that ferroportin protein levels peak at 100 μM of added ascorbate with an increase of 274% (*p* = 0.02). At 150 μM of ascorbate, the increase was only 28% (*p* = 0.04), and at 200 μM there was no significant change from the baseline control. In addition, the ascorbate-induced, (at 150 μM) up-regulated ferroportin levels were associated with increased ^55^Fe transport across the basolateral border (19%, *p* = 0.03). Ascorbate-induced up-regulation of cellular ferroportin levels (no added iron) was associated with increased levels of the iron regulatory protein IRP2 (230%, *p* = 0.0009), and the hypoxia-inducible factor HIF2α (69%, *p* = 0.03). Thus, iron transport across the basal border via ferroportin is influenced by the intracellular status of ascorbate and IRP2 and HIF2α are involved. We discuss possible reasons for the ascorbate-effects and the dependence of cellular growth conditions for iron transport-related protein expression.

## 1. Introduction

Ascorbate, the anionic form of ascorbic acid (vitamin C), is an important mediator in several cellular processes where it mostly functions as an electron donor in enzymatic reactions. Two important functions of ascorbate are to serve as a cofactor for hydroxylases required for the stabilization of collagen in skin [1] and the formation of the catecholamine norepinephrine [2] in the adrenal glands. Ascorbate is a well-known enhancer of nutritional iron uptake in the human intestine [3] and in human intestinal cell lines [4,5]. Until recently, this enhancement was thought to simply be a lumenal effect due to the reduction of ferric iron to ferrous iron by ascorbate. Ascorbate has been shown to possess pH-dependent chelating properties [6], forming soluble complexes [7], that could facilitate iron transport in the intestinal lumen. Recent studies show that ascorbate has intracellular effects on iron transporters and that iron increases the cellular uptake of ascorbate [8]. We have shown that intracellular ascorbate status affects the iron uptake related proteins DMT1 (NRAMP2) and DCYTB in human intestinal Caco-2 cells [9]. We also observed that approximately 50% of the increase in the intracellular iron storage protein ferritin was due to replete intracellular ascorbate levels. Another important observation was that expression levels of DMT1 and DCYTB were time dependent, showing an initial up-regulation and a later down-regulation. These data support the results of human studies, which indicate that continuous administration of ascorbic acid to the diet does not improve iron status [10]. In the light of these previous studies, it is convincing that ascorbate plays an important role in the absorption of iron that requires further elucidation in order to make use of the beneficial effects of ascorbic acid in human nutrition [11].

In the present study, we investigated the effects of ascorbate on the transfer of iron across the basal border of human intestinal Caco-2 cells. We also examined the cellular levels of the iron transporter ferroportin (FPN1/IREG1/SLC40A1), which is believed to be the main responsible transporter for the transport of ferrous iron (Fe^2+^) across the basal membrane of enterocytes into the portal circulation [12]. Ferroportin mRNA contains an iron response element (IRE) motif and like DMT1, ferritin, and the transferrin receptor, ferroportin is subject to iron-dependent regulation by the interaction of iron regulatory proteins (IRPs) with the IRE sequence [13]. Thus, ferroportin mRNA expression is regulated by the intracellular iron concentration. At present, there are two different IRPs identified, IRP1 and IRP2, which respond differently to iron. When cytosolic iron levels are low, IRP1 binds to IRE-motifs and either represses or promotes translation of IRE-containing mRNA. However, when intracellular iron levels are high, the IRE binding site of IRP1 associates with an iron-sulfur cluster and thus changes the IRE-binding site into an aconitase active unit [14] with no affinity for IREs. IRP2 does not convert into an aconitase. Instead, when iron levels are high, IRP2 is degraded by an ubiquitin ligase protein complex [15]. This complex contains an iron-responsive domain that is crucial for IRP2 degradation [16]. It is clear that the action of IRPs is very important in regulating the mRNA and protein levels of many iron absorption-related proteins. Since several of these proteins also appear to be regulated by ascorbate, we were eager to investigate additional factors. Here, we suggest that IRP2 and HIF2α are involved in the regulation of basolateral transport of iron through ferroportin in response to ascorbate in the human intestinal Caco-2 cell model. We also compare the difference in protein expression depending on if the cells were grown on a permeable filter support or attached to an impermeable plastic surface. Both these methods are frequently used, but unfortunately give different results.

## 2. Materials and Methods

### 2.1. Materials

Polystyrene disposables for cell cultivation were produced by Corning (San Fransisco, MA, USA). Media, supplements, and other reagents for culture maintenance were obtained from PAA (Pasching, Austria). Reagents used for the cell experiments were bought from Sigma Aldrich (Schnelldorf, Germany). All components for the enzyme-linked immuno-sorbent assay (ELISA) analyses, except for the primary antibodies, were purchased from Invitrogen (Paisley, UK). Ascorbate was used in the form of sodium ascorbate and iron was added as Fe(II)Cl_2_·4H_2_O labeled with ^55^FeCl_3_.

### 2.2. Antibodies

All antibodies were commercially produced, tested, and recommended for the application. The primary capture antibodies in the ELISA analyses were rabbit anti-human ferroportin peptide (ab58695; Abcam, Cambridge, UK), rabbit anti-human IRP2 (LS-C80347, LifeSpan Biosciences, Seattle, WA, USA), rabbit anti-human HIF2 (ab73895; Abcam, Cambridge, UK). Mouse anti-human Immunoglobin G (IgG) conjugated with horseradish peroxidase (HRP; #55788; BD, Franklin Lakes, NJ, USA) was used as secondary capture antibody. The primary antibody used in the Western blot analyses was a rabbit anti-human ferroportin (NBP1-21502; Novus Biologicals, Littleton, MA, USA). A rabbit anti-human β-actin antibody (ab8227; Abcam, Cambridge, UK) was used as loading control. For Western blot analyses, the secondary antibody was goat anti-rabbit IgG conjugated to HRP (Bio-Rad, Sundbyberg, Sweden). Primary and secondary antibodies were used at 1 μg/mL.

### 2.3. Cell Line

Caco-2 cells (HTB-37) were obtained from the American Type Culture Collection (Rockville, MD, USA) at passage 20. Stock cultures were maintained in Dulbecco’s modified Eagle’s medium supplemented with fetal bovine serum (FBS; 16%) and non-essential amino acids (0.9%). The medium contained an agent for the prevention of bacterial, fungal, or mycoplasma infection; Normocin (Invivogen, San Diego, CA, USA). The cell cultures were incubated at 37 °C in 95% humidified air and 5% CO_2_. The medium was changed every second or third day and the cells were passaged at approximately 80% confluence. At passage 29–39, the cells were seeded in 12-well plates with Transwell^®^ polycarbonate inserts (Corning, San Fransisco, MA, USA (0.4 μm) at 60,000 cells/insert or without inserts at 200,000 cells/well. All experiments were carried out 14 days after seeding. Aspirated medium samples were streaked on tryptone glucose extract agar plates to verify the absence of bacterial/fungal infections.

### 2.4. Cell Culture and Iron-Uptake Experiments

Thirteen days after seeding, the basal medium was exchanged for minimal essential medium (MEM) with Earle’s salt (E15-825; PAA, Pasching, Austria) supplemented with 2% FBS. The apical medium was changed to unsupplemented MEM. The cells were treated with sodium ascorbate by adding 5 μL of a stock solution to the basal or apical medium to obtain the final concentrations of 0, 100, 150, 200, 300, or 400 μM). The iron-uptake experiments were conducted on day 14 after 24 h of ascorbate supplementation. The apical medium in all plates was exchanged for freshly prepared MEM, unsupplemented or supplemented with iron. The iron concentration in apical chambers was 20 μM and the cells were incubated with iron for 2 h during agitation (25 rpm; 37 °C). The integrity of the monolayers was verified by trans-epithelial electrical resistance (TEER) measurements to ensure that the epithelial layers were intact.

### 2.5. Harvesting of Caco-2 Cells for Protein Analysis

Prior to harvest, the medium was aspirated and the cells were washed in PBS. Cells were lysed in cold RIPA buffer (Sigma Aldrich, Schnelldorf, Germany) containing an EDTA-free protease inhibitor cocktail (40 μL/mL) (Roche, Basel, Switzerland). Total cellular protein content was estimated by the bicinchoninic acid assay (Pierce, Chicago, IL, USA).

### 2.6. Sandwich ELISA for Estimation of Ferroportin, IRP2, and HIF2α Protein Expression

The Amplex^®^ ELISA development kit (Invitrogen, Paisley, UK) was used according to manufacturer’s protocol, with modifications. Briefly, microplates were coated with capture antibodies (rabbit anti-human IRP2, HIF2α, and ferroportin) at 1 μg/mL (overnight, 4 °C). The plates were washed in PBS tween and blocked in PBS-BSA 1% (overnight, 4 °C). After washing, 10 μL of sample in PBS-BSA (0.1%) was added to each well for incubation (1 h). The wells were washed once again and, incubated with the secondary capture antibody (mouse-anti-human IgG-HRP) for 30 min. After the final washing step, the wells were added the reaction mixture containing the Amplex^®^ UltraRed reagent. The signal was detected after 30 min with a fluorescence plate reader (excitation wavelength 530 nm/emission wavelength 590 nm; Tecan GmbH, Salzburg, Austria). The fluorescence gain from the samples was normalized to the total protein concentration in each sample.

### 2.7. SDS-PAGE and Western Blot for Detection of Ferroportin Levels

Cell lysates were diluted in 2× Laemmli sample buffer with β-mercaptoethanol and boiled at 95 °C for 5 min. Samples (20 μg protein) were loaded on TGX-gels (any kD; Bio-rad, Sundbyberg, Sweden) and electrophoresed with Tris/glycine/SDS buffer at 200 V. The molecular weight standard was a Precision Plus Protein Western C standard. After electrophoresis, the separated proteins were blotted to a polyvinyl difluoride (PVDF) membrane using the Trans-Blot Turbo system with pre-packed transfer packs and the 3-min protocol (Bio-rad). The blots were incubated in blocking buffer (Sigma Aldrich, Schnelldorf, Germany) at room temperature for 1 h. The primary antibody was diluted in blocking buffer (1 μg/mL) and the incubation lasted for 1 h or overnight at 4 °C. Then, the blots were washed in PBS-tween 20 (5 × 10 min, 25 rpm). The blots were incubated with secondary antibody (1 μg/mL) and a StrepTactin-HRP conjugate (2 μL) for 1 h. A strenuous washing protocol was used (5 × 10 min, 40 rpm). Finally, the blots were added a solution of luminol and peroxide buffer (Bio-rad) and the bands were detected by the ChemiDoc™ XRS+ system (Bio-rad) and analyzed with the software Image Lab™ 3.0.1 (Bio-rad).

### 2.8. ^55^Fe Transmembrane Transport

Caco-2 cells were cultured on permeable polycarbonate inserts as described in a former section, the only difference being that the medium contained no antibiotics. On day 13, 6 (or 2) sets of cells were either treated for 19 h with ascorbate (150 μM) in the basal chamber or left untreated before commencement of the iron-uptake studies. The cells were exposed to a solution of FeCl_2_ labeled with ^55^FeCl_3_ for 2 h (total iron was 20 μM and the activity was 5.4 Mbq/mL). ^55^FeCl_3_ was stabilized in nitrilotriacetate at a ratio of 1:4 before addition to the FeCl_2_ solution, which was allowed to equilibrate for 1 h before incubation with the cells. Uptake of ^55^Fe and transfer from the cells to the basolateral chamber, were measured by counting β-emission using a Tri-Carb 1900CA liquid scintillation analyzer (Packard Instrument, Meriden, CT, USA).

### 2.9. RNA Purification and cDNA Synthesis

Cellular RNA was purified using the RNeasy^®^ Mini Kit (Qiagen Gmbh, Hilden, Germany) according to the manufacturer’s instructions. An optional on-column DNase digestion was also conducted (Qiagen cat. no. 79254). Flow-throughs were discarded by vacuum assisted aspiration and the procedure was conducted under laminar airflow. In the last step, the columns were transferred to new collection tubes (Biopure; Sigma Aldrich, St. Louis, MO, USA) and RNase free water (50 μL) was added directly to the membrane. The RNA was eluted by centrifugation for 1 min at 8000 *g*. The samples were frozen in −80° until further analyses. The RNA concentration in samples diluted in Tris-HCl (10 mM, pH 7.0) was determined spectrophotometrically at 260 nm using a Tecan Sapphire II fluorescence microplate reader (Tecan Gmbh, Salzburg, Austria). The purity of the samples was evaluated by measuring the absorbance ratio of 260/280 nm. Single-stranded cDNA was synthesized using a commercial kit (Applied Biosystems, Foster City, CA, USA) according to the manufacturer’s instructions. The reverse transcription was performed using a thermal cycler (Bio-rad Gene cycler™, Hercules, CA, USA).

### 2.10. Real Time PCR Measurements of Ferroportin mRNA Expression

For real time PCR (qPCR), The TaqMan gene expression system was employed. TaqMan Gene Expression Master Mix (Applied Biosystems, Foster City, CA, USA) was used following the manufacturer’s instructions. The ferroportin primer pair and probe (ref number Hs00205888_m1) was selected together with the human GAPD (GAPDH, FAM/MGB Probe, Non-Primer Limited reference no.: 4333764F) as the internal control. Target and controls were run in separate wells. For each amplification, 1 or 3 ng cDNA was used in a reaction volume of 20 μL. For thermocycling, a Stratagene MX 3005P instrument (SA Biosciences, Frederick, MD, USA) was used. The PCR started with incubation at 95 °C for 10 min followed by 40 cycles of 95 °C for 15 s and 60 °C for 60 s. The fluorescence detection was done in each cycle at 60 °C. PCR-products were visualized on an ethidium-stained TAE-agarose gel (1%) using the Gel Doc 2000 equipment (Bio-rad, Hertfordshire, UK).

### 2.11. Statistics

Calculated values are presented as means ± standard deviation (SD). Cell experiments were made in triplicates and repeated at three occasions. Number of replicas are stated as *n* = x. The significance of the difference between treatment and control was analyzed by Student’s two-tailed, unpaired *t* test using Microsoft Office Excel 2010 or 2011. Differences were considered significant at *p* < 0.05.

## 3. Results

### 3.1. Ferroportin, IRP2, and HIF2α Levels Peaked at 100 μM of Ascorbate

**Figure 1:**
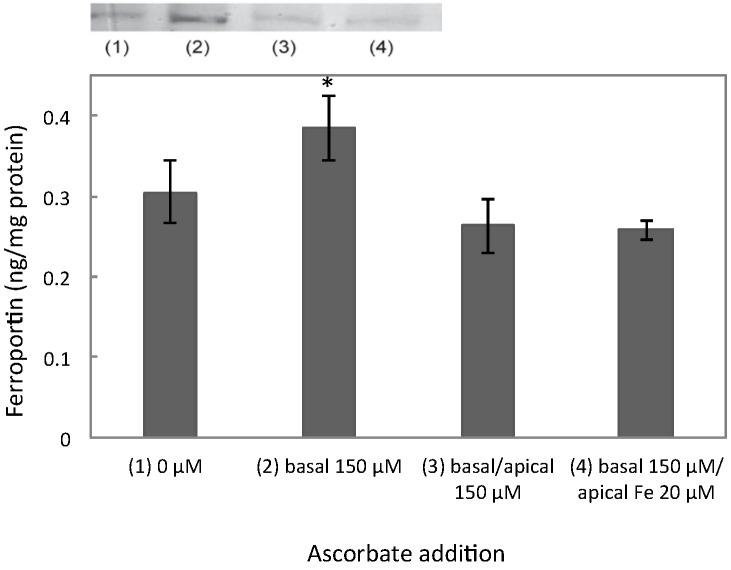
Sodium ascorbate (150 μM) was added to the apical and/or basal medium of Caco-2 cells cultured on permeable Transwell^®^ inserts. Ferroportin protein expression was measured with ELISA. Data are means ± SD, *n* = 12. Significant differences from the baseline (0 μM) are labeled with an asterisk (*). A western blot of ferroportin at the corresponding treatments is shown above the graph.

**Figure 2:**
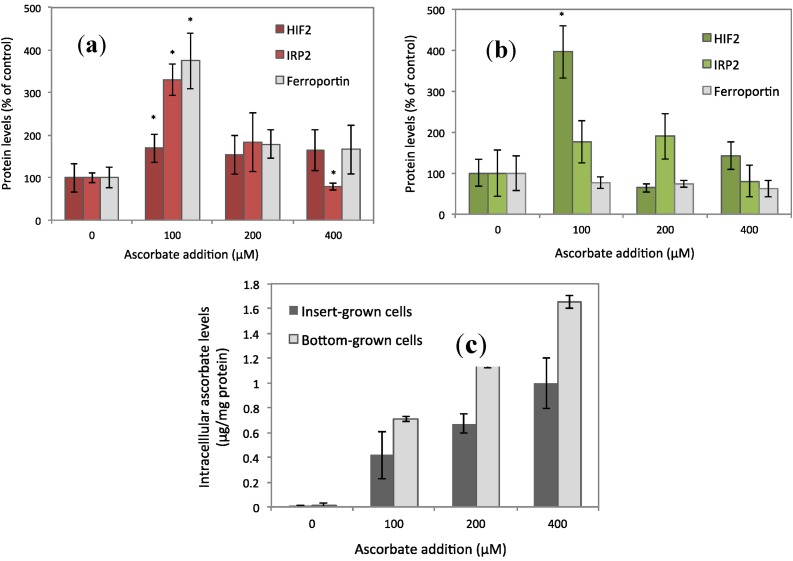
(**a**) Sodium ascorbate was added to the basal medium of Caco-2 cells cultured on permeable Transwell^®^ inserts. HIF2, IRP2, and ferroportin protein expression was measured with a sandwich ELISA. Data are means ± SD, *n* = 6. Significant differences from the baseline (0 μM) are labeled with an asterisk (*). (**b**) The same experiments as in (**a**) were repeated using Caco-2 cells cultured directly on the bottom of the wells. Instead of basal addition of sodium ascorbate, ascorbate was added to the apical medium. (**c**) Intracellular ascorbate levels of cells treated with sodium ascorbate (0, 100, 200, and 400 μM). Data are means ± SD, *n* = 3.

In the initial experiments we observed that enterocyte ferroportin levels were increased in the presence of ascorbate, added to the basal medium of membrane-cultured cells at 150 μM (28% ± 4%, *p* = 0.04, *n* = 12) and decreased in the presence of iron (20 μM) in combination with ascorbate (150 μM, Figure 1). We have also learnt that doubling the concentration of ascorbate (300 μM) down-regulates ferroportin levels (unpublished data) [17]. In the present work, we have therefore investigated ferroportin, IRP2, and HIF2α protein expression in a range of basally added ascorbate concentrations (0, 100, 200, and 400 μM). The results indicate that ferroportin levels peak at 100 μM ascorbate with an increase of 274% (*p* = 0.02, *n* = 6), as shown in Figure 2a. Increases were also observed for IRP2 (230%, *p* = 0.0009, *n* = 6) and HIF2α (69%, *p* = 0.03, *n* = 6). The levels of HIF2α at 200 and 400 μM ascorbate were insignificantly increased by 28% and 34% (*p* = 0.22 and *p* = 0.14, *n* = 6) from the baseline. In similar experiments, using Caco-2 cells cultured directly on the bottom of the wells, there was no observable difference in ferroportin levels at any concentration of ascorbate (Figure 2b). The IRP2 levels were not significantly increased at 100 μM (76%, *p* = 0.11, *n* = 6), in contrast to the insert-cultured cells. However, HIF2α levels were significantly amplified at 100 μM (295%, *p* = 0.02, *n* = 6), which may suggest that HIF2α is not as sensitive to enterocyte iron levels as ferroportin or IRP2, which will be discussed later on.

### 3.2. Increased Ferroportin Levels, Increased Transmembrane Fe Transport

**Figure 3:**
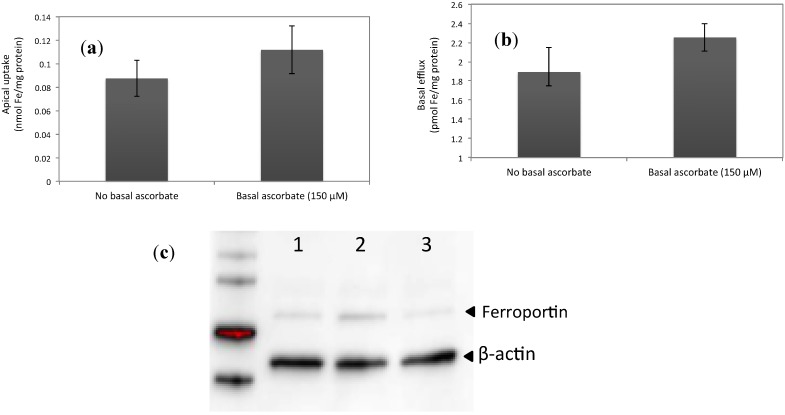
Iron (^55^Fe) transport across the apical and basal borders. (**a**) Apical transport (uptake) of ^55^Fe in response to intracellular/basal ascorbate (150 μM) was increased compared to no ascorbate. The cells were incubated with ascorbate in the basal chamber for 24 h. Values are means of 10 samples ±SD. The difference between treatments was significant (*p* = 0.04). (**b**) Basolateral transport of iron (as ^55^Fe) in response to intracellular/basal ascorbate (150 μM) was increased compared to no ascorbate. The cells were incubated with ascorbate in the basal chamber for 24 h. Values are means of 10 samples ±SD. The difference between treatments was significant (*p* = 0.03). (**c**) Western Blot of cells treated with ascorbate (150 μM). Lane 1: Control cells (no treatment). Lane 2: Basal ascorbate at 150 μM; these were the ferroportin levels before iron addition in (**a**). Lane 3: Ferroportin levels 22 h after the iron addition (20 μM for 2 h) in (**a**).

The cell lysates, basal, and apical media were analyzed for ascorbate/dehydroascorbate levels as described previously [9] using the method of Margolis *et al.* [18]. The intracellular ascorbate concentrations referring to Figure 2a,b are presented in Figure 2c. In the experiments at 150 μM of ascorbate (Figure 1 and Figure 3), the intracellular ascorbate levels in control cells were on average 0.8 ng/mg protein and in ascorbate-incubated cells (150 μM) the levels were on average 0.9 μg/mg protein. In the experiments, we used serum-free MEM in the apical medium and MEM-FBS 2% in the basal medium. We found that the apical medium was devoid of ascorbate/dehydroascorbate (data not shown), indicating that there was no transport of ascorbate from the cell interior to the apical surface. Thus, when iron was provided in the apical chamber and ascorbate in the basal chamber, there was no enhancement of iron uptake due to direct reduction of Fe^3+^ to Fe^2+^ by apical (lumenal) ascorbate. In these experiments, we investigated if increased ferroportin levels (30%, by ascorbate at 150 μM) had any functional implication in that iron transport would be facilitated. Ascorbate-replete cells were treated with apical iron (20 μM, 2 h) and iron uptake and basal transport was measured by means of radiolabeled iron (^55^Fe), which yielded a significant increase in iron uptake (28%; from 0.09 ± 0.015 to 0.11 ± 0.02 nmol Fe/mg protein, *p* = 0.04) and in basal transfer (19%; from 1.89 ± 0.26 to 2.25 ± 0.15 pmol Fe/mg protein, *p* = 0.03, *n* = 8, Figure 3a,b). The increase in iron uptake supports previous results, which showed that the apical iron transporter DMT1 (Nramp2) and the intracellular iron storage protein ferritin, were up-regulated in the presence of ascorbate [9]. The increase in basolateral transfer may be greater in reality, due to a possible underestimation of the transmembrane basal transport of iron using the present method [19]. The ferroportin protein levels were visualized by Western Blot before treating the cells with ascorbate (150 μM) and after ascorbate (150 μM) and iron (20 μM) incubation as shown in Figure 3c.

### 3.3. Ferroportin mRNA Levels Were Unchanged at 150 μM Ascorbate

We also measured the ferroportin mRNA expression in cells exposed to sodium ascorbate (150 μM for 24 h) and/or iron (20 μM for 2 h). The q-PCR analysis showed that there were only minor and statistically insignificant changes for all treatments (data not shown). To affect the RNA expression, more strenuous treatments seem to be required. In a study by Martini *et al.* [20], Caco-2 cells were exposed to ascorbate (1 mM) together with iron (200 μM) for 72 h. This treatment caused a down-regulation of ferroportin mRNA-levels.

## 4. Discussion

### 4.1. The Effects of Ascorbate—Just a Change in Redox Balance of the Cell?

Ascorbate is an important lumenal reducing agent for ferric iron (Fe^3+^) and copper (Cu^+^) to make these ions available for the cellular uptake proteins. However, ascorbate is also an important intracellular co-factor for the reductase DCYTB to which it distributes electrons to aid the enzymatic reduction of Fe^3+^ in the lumenal membrane of human enterocytes [21]. Ascorbate initially increases the cellular levels of DCYTB and iron uptake via DMT1, in addition to increasing the efflux of iron by ferroportin. Hypothetically, if the net cellular levels of iron would increase until steady state is reached, the excessive iron should be directed for incorporation in ferritin molecules (we have observed increases in ferritin levels), leaving “normal” iron levels in the labile iron pool (LIP). However, the redox state of iron in the LIP may thus have moved towards the reduced ferrous state (Fe^2+^) and the cells may sense the increase in Fe^2+^ as an increase in iron load, which may be a plausible explanation since ferroportin transports Fe^2+^ and not Fe^3+^. In fact, such an increase of Fe^2+^ in the LIP in ascorbate-replete, compared to ascorbate-deficient cells has been observed in brain cells (astrocytes) [22]. This would explain the down-regulation of IRP2 protein levels at higher concentrations of ascorbate. It would not explain the decreasing ferroportin levels while IRP2 is decreasing. If the ascorbate effect was only an increase in available iron, the degradation of IRP2 should instead promote ferroportin translation and thus increase its abundance. It seems more likely that ascorbate causes an iron-independent activation of the RNA-binding activity of IRP2 by the reduction of cysteine residues near the IRE-binding site [23], which blocks the translation of ferroportin mRNA. This results in a down-regulation of both IRP2 and ferroportin protein levels as observed here. That the ascorbate-induced down-regulation of ferroportin levels is a posttranscriptional event is also supported by the mRNA expression data, which suggested that ascorbate (150 μM) did not affect the cellular ferroportin mRNA content. Recently, it was shown that intracellular ascorbate facilitates uptake of transferrin-bound iron (Tf-^59^Fe) in cell types of non-enterocyte origin, and interestingly that it was independent of a general increase in cellular reducing capacity [24]. It remains to be evaluated if this is also true for intestinal absorptive cells. In summary, once the cells sense an increased iron load, increasing intracellular concentrations of ascorbate down-regulate ferroportin and its transport of iron. The initial increase of ferroportin levels from 0 to 100 μM of ascorbate is harder to explain; the effects we observe are the effects we would expect in iron-deficient cells. However, it seems unlikely that the cells would be more iron deficient at 100 μM of ascorbate compared to control cells with no ascorbate addition at all. The question is why all these iron-absorption associated proteins (DMT1, DCYTB, ferritin, ferroportin, IRP2, and HIF2α) increase with “low” concentrations of ascorbate?

### 4.2. Insights from Comparison of Permeable and Impermeable Basal Support for Cell Cultures

Cell monolayers cultured on permeable Transwell^®^ inserts have access to nutrients from both sides of the polarized cells. In addition, the absorptive enterocyte is specialized in transporting nutrients from the apical side across the cell and the basal membrane. It also responds to the systemic requirements of iron to increase or decrease apical to basal transport of iron. Translated into cell culture, this implicates that if an iron-free basal medium is used, systemic iron deficiency is implied and an apical to basal iron surge is induced. Similarly, if the cells are growing in high-iron medium or on an impermeable support, less or no apical to basal transport is facilitated, which traps iron inside the enterocyte. In the present work, we observed a distinct difference in cellular ferroportin levels in insert-cultured cells compared to bottom-grown cells. In cells growing on impermeable supports, ferroportin levels were unchanged, independent on ascorbate concentration. Also, the general expression of the investigated proteins, normalized to total cellular protein, was only a third of the expression in the insert-cultured cells. A similar trend for IRP2 levels as in insert-cultured cells could be anticipated, but the changes were insignificant for all ascorbate concentrations. In all, this may be interpreted as an inability of bottom-grown cells to respond fully to the increased iron load caused by ascorbate effects on DMT1 in combination with the entrapment. In contrast, HIF2α was significantly increased at ascorbate (100 μM) in both culture systems, suggesting that HIF2α is not as sensitive to the enterocyte intracellular iron concentration as ferroportin and IRP2. HIF2α has been shown to increase during iron deficiency in mice, and appears to be regulated by systemic iron load [25].

## 5. Conclusions

Our findings indicate that intracellular/basolateral ascorbate status regulates the transport of iron through ferroportin across the basal border of intestinal Caco-2 cells. The ascorbate effect, caused by the higher concentrations of ascorbate in the present work, may be a dual effect on IRP2, which expression decreases due to a sensed increase in iron load while the reducing capacity of ascorbate activates the iron-independent RNA-binding activity of IRP2, which blocks ferroportin translation. This results in down-regulation of both IRP2 and ferroportin protein levels as observed here. The initial up-regulating effect of ascorbate on ferroportin and IRP2 levels are likely to be the cause of cellular iron deficiency, which increases HIF2α levels. The increase in HIF2α up-regulates the ferroportin and IRP2 expression. However, we are at the moment not able to explain the peaking effect of ferroportin, IRP2, or HIF2α by ascorbate at 100 μM. Further studies are needed.
